# Assessment of depression and anxiety in Tunisian patients with chronic diseases: impact on quality of life and medication adherence

**DOI:** 10.3389/fpsyt.2025.1677506

**Published:** 2025-10-29

**Authors:** Cherif Farah, Masmoudi Rim, Frikha Chourouk, Abdelkefi Mariam, Guermazi Fatma, Emna Meziou, Feki Ines, Masmoudi Jawaher

**Affiliations:** ^1^ Psychiatry Department “A”, Hedi Chaker Teaching Hospital, Sfax, Tunisia; ^2^ Department of Family Medicine, Faculty of Medicine of Sfax, University of Sfax, Sfax, Tunisia; ^3^ Preventive and Community Medicine Department, Hedi Chaker University Hospital, Sfax, Tunisia

**Keywords:** chronic disease, anxiety, depression, quality of life, treatment adherence, North Africa

## Abstract

**Introduction:**

Chronic illness affects patients’ quality of life and often leads to underdiagnosed psychological issues, which can impact treatment adherence. This study aimed to assess quality of life in Tunisian patients with chronic diseases, screen for anxiety and depression, and evaluate their effect on medication adherence.

**Methods:**

We conducted a cross-sectional study including 170 patients consulting at the National Social Security Fund for chronic conditions, from September to November 2022. Quality of life was assessed using the Short Form 12 Health Survey, anxiety and depression using the Hospital Anxiety and Depression scale, and treatment adherence using the Morisky, Green, and Levine scale.

**Results:**

Among the 170 patients included in this study, 116 were women (68.2%), with a female-to-male ratio of 2.14. The mean age was 60 years and 5 months ± 9.85 years. The most common conditions were cardiovascular (71.7%), endocrine (64.7%), and pulmonary diseases (40.5%). The prevalences of depression and anxiety were 51.8% and 47%, respectively. Quality of life was impaired, with mean physical and mental scores of 35.05 ± 9.69 and 44.32 ± 11.13, respectively. Anxiety was more prevalent among women (p<0.001) and individuals under 60 years of age (p=0.009). Depression and anxiety were negatively correlated with quality of life. Depressive symptoms were associated with lower levels of treatment adherence (p=0.049).

**Conclusion:**

Anxiety and depression were frequent among patients with chronic diseases and were associated with poorer quality of life and lower treatment adherence. These findings underscore the burden of psychological distress in this population and highlight its detrimental effects on both well-being and disease management.

## Introduction

According to the World Health Organization, noncommunicable diseases (NCDs)—also commonly referred to as chronic diseases—are “long-term in duration and generally slow in progression,” resulting from a combination of genetic, physiological, environmental, and/or behavioral factors. It is a disease that generally causes physical, psychological, and/or cognitive impairment, imposing constraints and sometimes disabilities that affect quality of life (QoL), which represents a challenge for the patient. It is responsible for 74% of all deaths worldwide (1). In 2017, these NCDs were responsible for 87.7% of all disability-adjusted life years in Tunisia, with cardiovascular diseases, musculoskeletal disorders, neoplasms, and mental and neurological conditions ranking among the top contributors (2, 3).

In clinical practice, psychiatric disorders, particularly anxiety and depression, are often underdiagnosed and undertreated, masked by somatic therapeutic issues that take precedence (4). It is now clear that there is significant comorbidity between mood disorders, particularly anxiety and depression, and chronic conditions. This correlation is even more worrying given that this comorbidity profoundly undermines treatment adherence, increases morbidity and mortality, and complicates medical follow-up (5–8).

Several Tunisian studies focusing on single chronic conditions have examined the relationship between specific chronic diseases—such as diabetes, hypertension, or chronic respiratory diseases—and mental health status; however, they largely remain disease-specific in scope (9).

While previous studies have established the association between anxiety, depression, quality of life, and treatment adherence in specific chronic diseases such as type 2 diabetes, advanced lung cancer, or chronic kidney disease (6, 7, 10–13), our study is original in several respects. First, it is the first Tunisian investigation to examine these relationships collectively across a broad spectrum of chronic conditions in the primary care setting, thereby reflecting real-world multimorbidity rather than a disease-specific approach. Second, it addresses a major regional evidence gap, as data from North African countries remain scarce. Finally, it situates this analysis in the post-COVID socioeconomic context, where instability and limited access to mental health services are likely to exacerbate psychological distress and compromise adherence. By doing so, our study provides novel insights into the psychological burden of chronic illness in a resource-limited setting and highlights the need for integrated mental health strategies in routine chronic disease management.

Although the associations between depression, anxiety, quality of life, and treatment adherence have been widely investigated in different populations, evidence from North Africa, and particularly Tunisia, remains scarce. The Tunisian context presents specific cultural, socioeconomic, and healthcare-related characteristics that may shape these relationships in unique ways. For example, stigma surrounding mental health disorders, the central role of family support in coping with chronic illness, variations in socioeconomic status, and disparities in access to healthcare services may all influence both the expression of psychological symptoms and adherence behaviors. Generating data from this setting is therefore essential, not only to inform local health strategies but also to contribute to cross-cultural comparisons that may highlight differences and commonalities with international findings.

To the best of our knowledge, this is the first Tunisian study to examine a broad range of chronic conditions collectively while focusing on their association with psychological distress, particularly anxiety and depression, which remain underrecognized and undertreated in routine care.

This comprehensive approach is particularly relevant given the growing body of evidence showing that psychological distress significantly undermines treatment adherence, worsens clinical outcomes, and negatively affects quality of life in chronically ill populations.

In this context, our study aimed to assess the prevalence of anxiety and depression among patients with chronic diseases in primary care and to explore their impact on quality of life and medication adherence.

## Materials and methods

### Study type and population

This is a cross-sectional, descriptive, and analytical study conducted over three months, from September to November 2022, among patients followed at the polyclinic of the National Social Security Fund in Sfax.

We included patients over the age of 18 who had been followed for one or more chronic diseases for at least one year. We excluded patients who were already receiving psychiatric care and/or who had been hospitalized during the previous three months.

The minimum necessary sample size was 166 patients. It was calculated with an online program and estimated based on data from a Tunisian study (3). To estimate the minimum sample size needed in this study, we used an online program (OpenEpi program) with the following formula:


$$n=\frac{NZ^{2}pq}{\left(e^{2}\left(N-1\right)+Z^{2}pq\right)\;}$$

n=sample size; N=population size; Z=the statistic corresponding to level of confidence; p=proportion of total disability-adjusted life year in Tunisia for chronic diseases = 87.7% (3), q=1-P; e=precision. The estimation was also based on a 5% margin of error and a 95% confidence interval.

### Study procedure

Patients were recruited from the polyclinic of the National Social Security Fund in Sfax during routine medical consultations for chronic disease management. After their consultation, eligible patients were informed about the study by their treating physician and invited to participate. Those who expressed interest were referred to a trained physician-investigator, who provided further details and obtained verbal informed consent.

Data were collected through individual, face-to-face interviews, all conducted by the same physician to minimize interviewer variability.

All assessment instruments were administered during a single session, and each interview lasted approximately 20–30 minutes. Participation was voluntary, and no financial compensation was offered. Refusals were not systematically recorded; however, they were infrequent and generally due to time constraints.

### Data collection

We used a pre-established hetero questionnaire to collect sociodemographic data, medical history, type of pathology, and current treatments during interviews conducted by the same physician, ensuring consistency and reliability of information. In addition to this questionnaire, we used three psychometric assessment scales.

The validated Arabic versions of the Hospital Anxiety and Depression Scale (HADS), the Short Form-12 (SF-12), and the Morisky, Green, and Levine (MGL) adherence scale were used. Internal consistency was assessed using Cronbach’s alpha for each instrument.

The Cronbach’s alpha values were 0.82 for the HADS, 0.79 for the SF-12, and 0.74 for the MGL adherence scale, indicating acceptable to good internal consistency in our sample.

### Hospital anxiety and depression scale

Anxiety and depressive symptoms were assessed using the Arabic version of the Hospital Anxiety and Depression Scale (HADS) (14). This instrument, consisting of 14 items scored from 0 to 3, is used to screen for anxiety and depressive disorders. Seven questions concern anxiety (score A), and the remaining questions concern depression (score D). The scores are interpreted as follows: a score ≤7 indicates the absence of symptoms, a score between 8 and 10 suggests a possible state of anxiety or depression, and a score >10 indicates a definite state of anxiety or depression. For this study, we used a cut-off score of 7 to identify the presence of anxiety or depressive symptoms, consistent with previous literature.

Reliability analysis using Cronbach’s alpha (α) coefficient revealed good internal consistency for the Arabic HADS (*α* = 0.89) and both subscales (*α* = 0.86 for depression and *α* = 0.78 for anxiety) (15).

### Short Form 12 Health Survey

The SF-12 test is a shortened and validated version of the Medical Outcomes Study Short-Form General Health Survey (SF-36), consisting of 12 questions (16). The SF-12 calculates two scores: a physical health quality of life score and a mental health quality of life score, where a higher score reflects a better quality of life. We chose the Arabic version, which has been validated among the general Tunisian population (17), with a Cronbach’s alpha coefficient for the PCS-12 and MCS-12 scores of 0.76 and 0.74, respectively, which indicated satisfactory results (17).

### Morisky, Green, and Levine adherence scale

The level of therapeutic adherence was assessed using the Arabic version of the Morisky, Green, and Levine (MGL) adherence scale (18), which consists of 4 items, with a score of 0 for a “no” answer and 1 for a “yes” answer. The lower the score, the higher the level of medication adherence.

The Arabic language adopted in this version is the official language for multiple countries in the MENA region, with a Cronbach’s alpha for the 4 items = 0.593.

### Statistical analysis

The data collected was analyzed using the Statistical Package for Social Sciences (SPSS) software, version 20. Quantitative variables were expressed as means and standard deviations. Qualitative variables were expressed as frequencies. The relationship between qualitative variables (such as sex, marital status, education level, employment status, and Morisky score classes) was studied using Pearson’s chi-square test and Fisher’s exact test. The relationship between quantitative variables (such as age, HAD scores for depression and anxiety, and scores of the quality of life) was studied using Pearson’s correlation test. We used a significance threshold of p< 0.05.

### Ethical considerations

To ensure the ethical integrity of our study, several key safeguards were implemented:

- The study was conducted with the formal approval from the Ethics Committee of the Faculty of Medicine of Sfax (approval number: 69/25). All methods were performed in accordance with the relevant guidelines and regulations.
- We obtained permissions from relevant department heads before initiating data collection.
- The process of obtaining consent was adapted to the study context: since most participants were of advanced age and the questionnaire was administered by an interviewer (hetero-questionnaire), informed consent was obtained verbally. The interviewer explained clearly to each participant: the purpose, objectives, and framework of the study; the participant’s right to refuse to participate or to withdraw at any time without consequences, and the measures taken to ensure total anonymity and confidentiality.
- Where possible and when the participant was able and willing, written consent was also obtained.
- All data were treated with strict confidentiality and anonymized to ensure professional and ethical standards were maintained.
- Any participant screening positive for a psychiatric disorder was offered appropriate follow-up care, ensuring ethical responsibility toward participant welfare.

## Results

### Sociodemographic and clinical characteristics of the study population

Our study included 170 patients with a mean age of 60.5 ± 9.85 years, ranging from 32 to 79 years. The study population included 68.2% women (n=116), with a sex ratio (F/M) of 2.14. The majority of patients were married (83.5%, n=142), lived with their families (87.6%, n=149), and were of urban origin (84.7%, n=144).

Regarding education, 15.9% (n=27) of patients never received formal education, and 51.8% (n=88) had completed primary school. In terms of employment, 19.4% (n=33) were working at the time of the study, and 1.2% (n=2) were unemployed. An average socioeconomic level was reported by 69% of participants.

A majority of patients (74.7%) presented with multiple comorbidities, mainly cardiovascular (71.7%), endocrinological (64.7%), and pulmonary (40.5%) conditions. The average follow-up period was 13.1 years, and patients took an average of four medications per day. Regarding family history, 75% reported medical conditions, 24% reported surgical conditions, and 14.1% reported psychiatric conditions.

### Clinical psychometric assessment

According to the HADS scale, the average scores were 7.86 ± 4.66 for depression and 7.6 ± 4.52 for anxiety. Depressive symptoms were found in 51.8% of patients, while 47% presented with anxiety symptoms.

For quality of life, the average physical score was 35.05 ± 9.69 with a minimum of 18.84 and a maximum of 56.78, and the average mental score was 44.32 ± 11.13 with a minimum of 22.09 and a maximum of 68.33.

According to the Morisky score results, 36.5% of patients (n=62) had a high level of treatment adherence.

### Links between depression, anxiety, and sociodemographic and clinical factors

In our study, anxiety was correlated with female gender and age under 60 (**Table 1**).

**Table 1:** Sociodemographic factors influencing anxiety and depression levels among included patients (N = 170).

Sociodemographic factors		Depression p	Anxiety p
Age	≤ 60 (ref)	0.542	0.009*
	>60		
Gender	Female (ref)	0.138	<0.001*
	Male		
Origin	Urban	0.200	0.397
	Rural		
Marital status	Married	0.840	0.301
	Not married		
Living arrangement	alone	1.000	0.060
	With family/in an institution		
Profession	Presence of professional activity	0.702	0.567
	absence of professional activity		
Education	No formal education	0.681	0.532
	Educated		
Socioeconomic level	Low	0.462	0.273
	Average to high		

*Statistically significant association (ref): reference variable. Bold values are statistically significant association (p < 0.05).

We observed a higher prevalence of depressive symptoms in groups monitored for endocrine diseases. Patients with a history of rheumatic diseases had a higher prevalence of anxiety symptoms (**Table 2**). As for correlations, a very weak positive correlation was observed between the number of treatments received per day and anxiety and depression (r = 0.005 and r = 0.008, respectively), both of which were not statistically significant (p = 0.949 and p = 0.923).

**Table 2:** Clinical factors influencing anxiety and depression levels among included patients (N = 170).

Clinical factors		Anxiety p	Depression p
Cardiovascular conditions	Absent	0.733	0.060
	Present		
Endocrinological conditions	Absent	1.000	0.004*
	Present		
Pulmonary conditions	Absent	0.278	0.161
	Present		
Nephrological conditions	Absent	0.667	1.000
	Present		
Gastroenterological conditions	Absent	1.000	0.369
	Present		
Rheumatological conditions	Absent	0.035*	0.834
	Present		
Neurological conditions	Absent	0.150	1.000
	Present		
Other	Absent	0.685	0.683
	Present		
Follow-up duration (years)	< 5	0.188	0.352
	≥ 5		
	< 2	0.006*	0.073
	≥ 2		
Family history of psychiatric illness	Absent	0.512	0.060
	Present		

*Statistically significant association. Bold values are statistically significant association (p < 0.05).

### Links between depression, anxiety, and quality of life

Physical and mental QoL were significantly reduced in subjects with higher levels of depression. Similarly, the more anxiety symptoms increased, the more physical and mental QoL decreased (**Table 3**).

**Table 3:** Anxiety and depression levels among included patients according to their SF-12 scores.

SF-12 score		Anxiety score	95% confidence interval for anxiety score	Depression score	95% confidence interval for depression score
Physical score	R	-0.272	[–0.406; –0.127]	-0.387	[–0.508; –0.251]
	p	0.000*		0.000*	
Mental score	R	-0.712	[–0.779; –0.629]	-0.666	[–0.742; –0.573]
	p	0.000*		0.000*	

*Statistically significant association. Bold values are statistically significant association (p < 0.05).

### Links between treatment adherence and socio-demographic and clinical factors

In our study, we found no statistically significant correlations between treatment adherence and patients’ sociodemographic characteristics. However, patients with a history of endocrine diseases showed better treatment adherence (p=0.030).

### Links between levels of treatment adherence and quality of life

Patients with a high level of treatment adherence had higher mental and physical scores, reflecting a better quality of life (**Table 4**).

**Table 4:** SF-12 physical and mental scores among included patients according to their level of treatment adherence (N = 170).

Level of treatment adherence	SF-12	
	Physical score	Mental score
Low/Moderate	34.16	42.94
High	36.59	46.71
Mean difference and 95% CI	**+2.43** **[**–0.62 to +5.48]	+3.77 [+0.27 to +7.27]
p	0.117	0.034*

*Statistically significant association. Bold values are statistically significant association (p < 0.05).

### Links between treatment adherence and anxiety and depression

Patients with depressive symptoms had a lower level of treatment adherence (p=0.049) (**Table 5**).

**Table 5:** Level of treatment adherence according to depressive and anxiety symptoms, N = 170.

Anxiety and depression symptoms		Level of treatment adherence		p
		Low/Moderate	High	
Depressive symptoms	Absent	46	36	0.049*
	Present	62	26	
Anxiety symptoms	Absent	54	36	0.341
	Present	54	26	

*Statistically significant association. Bold values are statistically significant association (p < 0.05).

## Discussion

The present study assessed the prevalence of depression and anxiety in Tunisian patients with chronic diseases and examined their impact on quality of life and medication adherence. Our findings are consistent with international literature showing that psychological distress is common among patients with chronic conditions and is associated with poorer outcomes. However, the added value of this study lies in providing data from Tunisia, a country where research on the mental health of chronically ill patients remains limited. The Tunisian cultural and socioeconomic context – characterized by limited access to specialized mental health services, strong family involvement in health-related decisions, and persistent stigma toward psychiatric disorders – may influence both the recognition of symptoms and patients’ willingness or ability to adhere to treatment. By situating these findings within the local context, this study contributes to a better understanding of how depression and anxiety affect chronic disease management in underrepresented regions and opens avenues for culturally adapted interventions.

Our study showed a high prevalence of depression among patients with chronic diseases, exceeding the rates observed in global surveys (19). Indeed, the presence of chronic diseases appears to be a risk factor for depression (20).

The complex, bidirectional relationship between chronic conditions and depression is explained by various physiological and psychological mechanisms (21). On the one hand, depression can make individuals more vulnerable to diseases. It is associated with a prolonged activation of the sympathetic nervous system, the hypothalamic-pituitary axis, and the neuroendocrine system, which can lead to chronic inflammation, immune dysfunction, and hormonal changes (22). On the other hand, depression can develop after the diagnosis of chronic disease through several physiological mechanisms, such as specific biological mechanisms involved in certain physical conditions or psychological mechanisms (23).

In our study, there was a 25.9% prevalence of anxiety among patients with chronic diseases, which is higher than that observed in developed countries (22). This difference can be explained by the low socioeconomic status, difficulties in accessing healthcare, and the limited availability of essential treatments for chronic diseases in a lower-middle-income country with unstable economic conditions, such as Tunisia (24). These factors contribute to difficulties in receiving optimal care and promote the onset of anxiety symptoms compared to developed countries (25).

According to the literature, this relationship between anxiety and chronic physical illnesses can be attributed to several mechanisms. First, living with a chronic illness leads to constant and significant fears about the progression of the illness, hospitalizations, treatment outcomes, and checkups (26). Second, certain chronic diseases cause changes in the body, such as hormonal imbalances. Thus, these disease-related changes can then be expressed in the central nervous system through alterations in the ventral striatum and orbitofrontal cortex, dysfunction of the anterior insula and ventromedial prefrontal cortex, and hyperactivity of the amygdala (26).

Our study also highlighted a significant deterioration in the quality of life of patients with chronic diseases, with physical scores being particularly affected. Our population’s physical score was lower than the general population’s (47.8 ± 9.8), but the mental score was nearly identical to the Tunisian population’s results (27). These results are consistent with those reported in the literature. In a cross-sectional study conducted across six cities in Hubei Province, China, researchers collected 1,507 valid responses, of which 354 participants (23.5%) reported having one or more chronic diseases. The average age of the sample was approximately 53 years. Quality of life was assessed using the SF-12 Health Survey, yielding both Physical Component Summary (PCS) and Mental Component Summary (MCS) scores. The results showed that individuals with chronic conditions had significantly lower PCS and MCS scores—averaging 59.13 ± 20.17 and 71.14 ± 13.55, respectively—compared with healthy counterparts (28). Chronic diseases affect various aspects of patients’ lives, such as their sense of independence, social relationships, financial stability, and overall well-being, which are not usually assessed systematically during medical consultations. As a result, patients no longer feel capable of performing their usual tasks, which affects their mental health, generating feelings of sadness and helplessness.

### Links between depression and sociodemographic and clinical factors

We did not find any statistically significant correlations between depressive symptoms and sociodemographic factors.

However, women had a higher average depression score than men, although this difference was not significant. This is consistent with the literature, where female gender is correlated with depression (29, 30). Several theories can explain this relationship. Indeed, certain biological factors, such as hormonal changes, pregnancy, and menopause, contribute to women’s biological vulnerability to depression (31). Among premenopausal women, mood disorder symptoms may fluctuate across menstrual cycle phases, with many experiencing premenstrual exacerbations of depressive and anxiety symptoms (32). In older women, the perimenopausal period—characterized by declining and erratic estrogen levels—has been associated with increased risk of both anxiety and depression, notably during the late perimenopause “window of vulnerability” (33). The lack of a statistically significant sex difference in depressive symptoms in our study may be partly explained by the fact that many of the women in our sample may be in the perimenopause or menopause stage. Another hypothesis is the social discrimination suffered by women, in addition to the feelings of powerlessness and dependence on others, which can lead to symptoms of depression (31).

In our study, we found a significant correlation between depression and a history of endocrine diseases. Furthermore, the results did not show a significant correlation between the prevalence of depression and other clinical factors. The correlation between depression and endocrine diseases has been established in the literature. Fava et al. (34) have shown that depressive disorders can be a major complication of endocrine diseases such as Cushing’s syndrome, thyroid disorders, Addison’s disease, amenorrhea, and diabetes. The relationship between endocrine disease and depression appears to be bidirectional. The direct effects of hormonal imbalance, insulin resistance, and inflammation on neural structures, such as the limbic system, are mediators of damage to the central nervous system, leading to mood disorders and cognitive impairment. Conversely, depression induces hormonal changes, particularly in adrenal and thyroid hormones, which in turn induce the onset of endocrine diseases (34).

In our study, we did not find a significant relationship between cardiovascular disease and depression. However, more than half of patients with cardiovascular disease presented with depressive symptoms (56.5%). According to the literature, depression and cardiovascular diseases are considered mutual risk factors (35). A study conducted in Tunisia among patients attending the outpatient cardiology clinic following a myocardial infarction showed that “possible-to-definite” depressive symptoms were found in 68.1% of patients, and 63.8% presented with “possible-to-definite” anxiety symptoms (36). In the case of heart disease, for example, the association with depression could be explained by the fact that low cardiac output limits a heart failure patient’s activity and functioning, which may be associated with symptoms of depression (37). There is evidence linking platelet activation, autonomic activity, and inflammatory markers to both depression and heart disease. Similarly, depression is accompanied by some behaviors that will undoubtedly increase the risk of vascular disease: neglect of health, increased consumption of substances such as tobacco and coffee, and a sedentary lifestyle (35, 38).

We found a significant negative correlation between the SF-12 physical health score and the depression score, as well as between the mental health score and the depression score. Our results, and those in the literature, indicate a link between depression and poor health-related quality of life in patients with chronic diseases (39). It has been shown that patients suffering from both depression and chronic physical conditions have a more impaired QoL than those with chronic physical conditions who do not exhibit depressive symptoms (19). This relationship between chronic diseases and depression is mediated by health-related quality of life (40). Depression is thought to be an aggravating factor in the burden of chronic disease and individual suffering. The study by Stein, Murray B et al. (41) has shown that the presence of a major depressive disorder comorbid with chronic conditions is associated with a higher probability of disability and activity limitation compared to chronic disease without depressive comorbidity. Similarly, depression can impact QoL by impairing the patient’s cognitive and motivational abilities. It further reduces QoL by contributing to poor treatment compliance and complicating management (42). These repercussions typically exacerbate patients’ feelings of helplessness and powerlessness.

### Links between anxiety and sociodemographic and clinical factors

We found a significant correlation between the prevalence of anxiety symptoms and female gender. Our findings support those reported in the literature (43). Women are more affected by anxiety than men, which can be explained by hormonal factors, as well as their status and role in society. In fact, women are more likely to detect and report symptoms of anxiety.

We also found a significant correlation between the prevalence of anxiety symptoms and the age group under 60. The data in the literature are mixed on this point. Some studies, such as that by Camara et al. (43) in diabetic subjects, found that anxiety was more common in subjects under the age of 50. This higher prevalence in younger subjects could be explained in part by the stress factors they face and the particularities of this stage of life, notably the difficulty of maintaining a balance between work and family life (43).

Furthermore, we did not find any statistically significant correlations between the presence of anxiety symptoms and the other sociodemographic characteristics studied (origin, marital status, occupation, level of education, and socioeconomic status).

In our study, we found a statistically significant correlation between the prevalence of anxiety symptoms and rheumatic diseases. This finding is consistent with other studies in which authors have shown that patients with chronic rheumatic diseases are at greater risk of developing anxiety (39, 44). Rheumatic diseases are a major health problem. They are clinically diverse but are mostly characterized by a chronic and debilitating progression with significant long-term excess mortality. As a result, they have a significant psychological impact on patients’ lives. The pathogenesis of anxiety involves many interrelated factors, including genetic factors, changes in the central nervous system and autonomic nervous system, inflammatory alterations, and environmental factors (45). In addition, the presence of chronic pain caused by these rheumatic diseases is a contributing factor to the onset of anxiety symptoms (45).

Our results showed a significant negative correlation between the prevalence of anxiety and QoL. We found a significant negative correlation between the SF-12 physical score and the anxiety score, as well as between the SF-12 mental score and the anxiety score. Although this relationship has been little studied in the literature, the results highlight the same finding (44, 46). A German study highlighted the negative impact of anxiety comorbidity on the QoL of subjects suffering from somatic pathologies with no psychological distress (44). In the study by Lima et al. (46), among patients with chronic obstructive pulmonary disease, anxiety is associated with poorer outcomes in various areas of QoL. The presence of an anxiety disorder can reduce the ability to manage the disease, relapses, treatment adherence, repeated consultations, and other problematic situations. The patients thus find themselves caught in a vicious circle between deterioration in their QoL and psychological difficulties.

In light of these findings, several measures could be considered to improve the care of chronic patients. It would be essential to systematically integrate anxiety and depression screening into consultations and assessments for these patients. It is also crucial to raise awareness among physicians of the importance of mental health care, on a par with physical health care, to achieve the therapeutic goals of stabilizing chronic diseases.

### Factors associated with treatment compliance

In our study, the Morisky score showed that 21.2% of patients had a low level of treatment compliance, while 42.2% had a moderate level and 36.5% had a high level. Compared to developed countries, where the average compliance rate is 50% (47), our study shows a lower prevalence of good compliance. Factors such as doubts about the effectiveness of treatment, financial problems, lack of family support, and side effects of medication are identified as major barriers to good compliance (47). Other studies have found that patients with chronic diseases have difficulty understanding the information provided by healthcare professionals about their treatments, with a lack of understanding of their therapeutic objective (48).

We found a significant correlation between the prevalence of depressive symptoms and treatment adherence levels. Patients with depressive symptoms had lower levels of adherence. This finding is consistent with the results reported in the literature. Indeed, studies have shown that patients with depression have poorer treatment adherence (8). A meta-analysis conducted by DiMatteo et al. in 2000 showed that patients with comorbid depression were three times more likely not to adhere to treatment than patients without depression (49). There are several reasons why depression can affect treatment compliance. It is often associated with a decline in cognitive function, which is essential for remembering to take medication correctly. The social isolation experienced by depressed individuals may contribute to this forgetfulness. Additionally, depression is accompanied by despair, decreased motivation, and reduced willpower, all of which could affect patients’ habits, including their commitment to taking their medication regularly (50).

While the relationship between depression and treatment adherence is well-established, the relationship with anxiety has not been extensively studied. In the meta-analysis conducted by DiMatteo et al. (49), the relationship between treatment adherence and anxiety was not significant.

In general, anxiety and depression disorders can impair patients’ concentration, motivation, self-confidence, and energy. Patients then lack the will and ability to take their medication, creating a vicious cycle. This problem must, therefore, be taken into account to ensure better care and a better prognosis.

In our study, we found a statistically significant correlation between the level of treatment adherence and the mental score of the QoL. Thus, the group of patients with a high level of treatment adherence had a higher mental score. The results of our study did not show a statistically significant correlation between the level of therapeutic compliance and the physical score of the SF-12. Similar results to ours were demonstrated in the study by Hovinga et al. (51), where epileptic patients with poor compliance had lower SF-12 mental health scores than those with good compliance; however, they had similar physical scores.

Although existing literature increasingly highlights the bidirectional relationship between QoL and treatment adherence in patients with chronic diseases—showing that good adherence improves autonomy and daily functioning, while poor adherence exacerbates disease, complications, and declines in QoL (52)—there remains a notable gap in Tunisian primary care research. Few studies have investigated QoL and its impact on adherence among patients consulting for any chronic pathology in first-line care settings. Our study helps fill this gap by offering much-needed, real-world data reflecting the realities of primary care in Tunisia.

Therapeutic compliance should be assessed at each consultation. The integration of therapeutic education into patient follow-up is also essential to ensure good adherence to treatment and, consequently, better control of chronic diseases.

## Study limitations

One of our study limitations is its cross-sectional design, which implies that causality cannot be inferred from our findings. Thus, one main recommendation is to conduct prospective studies that ensure causality and temporality in the future. Our study focused on patients with chronic diseases who were followed up at the polyclinic of the National Social Security Fund. We chose this healthcare facility because its patient population is more diverse than that of specialized hospital services. However, due to logistical constraints, it was not possible to include all patients registered during the study period, as meeting eachindividual and obtaining informed consent proved challenging. Additionally, it should be noted that we used the Morisky, Green, and Levine (MGL) scale to assess adherence. While less widely employed internationally compared to the MMAS-8, theMGL has been validated in Arabic and offers a simple structure suitable for patients with different literacy levels. However, this choice may limit direct comparison with studies using MMAS-8, and future research could consider the latter to improve international comparability. Another limitation is the reliance on descriptive statistics and bivariate tests (chi-square and correlations) without multivariate adjustment. This approach does not account for potentialconfounding factors. Future studies with larger samples should incorporate logistic regression or other multivariate models to provide more robust evidence.

## Conclusion

This study underscores the critical interplay between chronic conditions and mental health among primary care patients in Tunisia. By systematically evaluating anxiety, depression, treatment adherence, and quality of life, our findings highlight a substantial gap in routine clinical practice—particularly the absence of integrated mental health screening and QoL assessment in patient management. Addressing this void is essential for achieving holistic, patient-centered care and enhancing both physical and psychological outcomes in chronic disease management.

## Data Availability

The original contributions presented in the study are included in the article/supplementary material. Further inquiries can be directed to the corresponding author.

## References

[1] World Health Organization (WHO). Noncommunicable diseases. Geneva: WHO. Available online at: https://www.who.int/news-room/fact-sheets/detail/noncommunicable-diseases (Accessed September 5, 2025).

[2] Guesmi N Ben Fredj S Zammit N Ghammam R Harrabi I Chouikha F. Intervention effectiveness in reducing the clustering of non-communicable disease risk factors in the workplace: A quasi-experimental study. PLoS One. (2025) 20:e0317460. doi: 10.1371/journal.pone.0317460, PMID: 39913562 PMC11801702

[3] Khiari H Mallekh R Cherif I Hsairi M. Burden of non-communicable diseases in Tunisia, 1990-2017: results from the global burden of disease study. Pan Afr Med J. (2021) 40:62. doi: 10.11604/pamj.2021.40.62.30980, PMID: 34804330 PMC8590256

[4] Abiodun OA. Mental health and primary care in Africa. East Afr Med J. (1990) 67:273−8. 2364903

[5] Alkaabi AJ Alkous A Mahmoud K AlMansoori A Elbarazi I Suliman A. The prevalence and correlates of depression among patients with chronic diseases in the United Arab Emirates. PLoS One. (2022) 17:e0278818. doi: 10.1371/journal.pone.0278818, PMID: 36516141 PMC9749973

[6] Abbas Q Latif S Ayaz Habib H Shahzad S Sarwar U Shahzadi M. Cognitive behavior therapy for diabetes distress, depression, health anxiety, quality of life and treatment adherence among patients with type-II diabetes mellitus: a randomized control trial. BMC Psychiatry. (2023) 23:86. doi: 10.1186/s12888-023-04546-w, PMID: 36737757 PMC9896442

[7] Fouda HDEEM Mouaffo FNM Nzana VB Tumchou P Elimby L Teuwafeu D. Household wealth and its association with non-adherence, quality of life, anxiety and depression amongst hemodialysis patients in two centers in Cameroon. BMC Nephrol. (2025) 26:447. doi: 10.1186/s12882-025-04380-0, PMID: 40783719 PMC12335032

[8] Hisan UK Widjanarko B Sriatmi A Shaluhiyah Z. Association between depression and medication adherence in noncommunicable diseases: a narrative review. Korean J Fam Med. (2025) 46:231−9. doi: 10.4082/kjfm.25.0018, PMID: 40716457 PMC12301678

[9] Rassas I Bel Hadj N Marmouch H Kilani W Merchaoui I Mahfoudh A. Predictors of physical and mental health-related quality of life in Tunisian workers with type 2 diabetes. J Occup Environ Med. (2025) 67:e642−8. doi: 10.1097/JOM.0000000000003449, PMID: 40480816

[10] Abbas U Hussain N Tanveer M Laghari RN Ahmed I Rajper AB. Frequency and predictors of depression and anxiety in chronic illnesses: A multi disease study across non-communicable and communicable diseases. PloS One. (2025) 20:e0323126. doi: 10.1371/journal.pone.0323126, PMID: 40333937 PMC12057975

[11] García-Llana H Remor E Del Peso G Selgas R. The role of depression, anxiety, stress and adherence to treatment in dialysis patients’ health-related quality of life: a systematic review of the literature. Nefrol Publicacion Of Soc Espanola Nefrol. (2014) 34:637−57. doi: 10.3265/Nefrologia.pre2014.Jun.11959, PMID: 25259819

[12] Akif A Qusar MMAS Islam M. The impact of chronic diseases on mental health: an overview and recommendations for care programs. Curr Psychiatry Rep. (2024) 26:394−404. doi: 10.1007/s11920-024-01510-7, PMID: 38767815

[13] Hwang Y Oh J. Relationship between depression, anxiety, stress, and health-related quality of life in adults with and without chronic diseases: A cross-sectional study. Med (Baltimore). (2024) 103:e36967. doi: 10.1097/MD.0000000000036967, PMID: 38215093 PMC10783309

[14] Malasi TH Mirza IA el-Islam MF. Validation of the hospital anxiety and depression scale in arab patients. Acta Psychiatr Scand. (1991) 84:323−6. doi: 10.1111/j.1600-0447.1991.tb03153.x, PMID: 1746281

[15] Ibrahim N Yamout D Bizri M Taher A. The Arabic Hospital Anxiety and Depression Scale: validation in a sample of Lebanese patients with cancer. Middle East Curr Psychiatry. (2023) 30:85. doi: 10.1186/s43045-023-00357-7

[16] Ware J Kosinski M Keller SD. A 12-Item Short-Form Health Survey: construction of scales and preliminary tests of reliability and validity. Med Care. (1996) 34:220−33. doi: 10.1097/00005650-199603000-00003, PMID: 8628042

[17] Younsi M. Health-related quality-of-life measures: evidence from Tunisian population using the SF-12 health survey. Value Health Reg Issues. (2015) 7:54−66. doi: 10.1016/j.vhri.2015.07.004, PMID: 29698153

[18] Awwad O AlMuhaissen S Al-Nashwan A AbuRuz S. Translation and validation of the Arabic version of the Morisky, Green and Levine (MGL) adherence scale. PloS One. (2022) 17:e0275778. doi: 10.1371/journal.pone.0275778, PMID: 36206237 PMC9543961

[19] Moussavi S Chatterji S Verdes E Tandon A Patel V Ustun B. Depression, chronic diseases, and decrements in health: results from the World Health Surveys. Lancet Lond Engl. (2007) 370:851−8. doi: 10.1016/S0140-6736(07)61415-9, PMID: 17826170

[20] Li H Ge S Greene B Dunbar-Jacob J. Depression in the context of chronic diseases in the United States and China. Int J Nurs Sci. (2018) 6:117−22. doi: 10.1016/j.ijnss.2018.11.007, PMID: 31406877 PMC6608796

[21] Stegmann ME Ormel J de Graaf R Haro JM de Girolamo G Demyttenaere K. Functional disability as an explanation of the associations between chronic physical conditions and 12-month major depressive episode. J Affect Disord. (2010) 124:38−44. doi: 10.1016/j.jad.2009.10.026, PMID: 19939461 PMC3659772

[22] Bica T Castelló R Toussaint LL Montesó-Curto P. Depression as a risk factor of organic diseases: an international integrative review. J Nurs Scholarsh Off Publ Sigma Theta Tau Int Honor Soc Nurs. (2017) 49:389−99. doi: 10.1111/jnu.12303, PMID: 28692781

[23] Dowrick C Katona C Peveler R Lloyd H. Somatic symptoms and depression: diagnostic confusion and clinical neglect. Br J Gen Pract J R Coll Gen Pract. (2005) 55:829−30., PMID: 16281997 PMC1570790

[24] Loukil F Zribi K Zribi E. Pénurie des médicaments: retour d’expérience d’un hôpital tunisien. Pharm Hosp Clin. (2020) 55:255−62. doi: 10.1016/j.phclin.2020.04.010

[25] Katoue MG Cerda AA García LY Jakovljevic M. Healthcare system development in the Middle East and North Africa region: Challenges, endeavors and prospective opportunities. Front Public Health. (2022) 10:1045739/full. doi: 10.3389/fpubh.2022.1045739/full, PMID: 36620278 PMC9815436

[26] Grupe DW Nitschke JB. Uncertainty and anticipation in anxiety: an integrated neurobiological and psychological perspective. Nat Rev Neurosci. (2013) 14:488−501. doi: 10.1038/nrn3524, PMID: 23783199 PMC4276319

[27] Chakroun M. Inequality and Social Heterogeneity in Self-Reported Health Status in the Tunisian Population: An Analysis Using the MIMIC Model (2014). Rochester, NY: Social Science Research Network. Available online at: https://papers.ssrn.com/abstract=3459679 (Accessed September 5, 2025).

[28] Lei X Ferrier JA Jiang H. Quality of life and associated factors among people with chronic diseases in Hubei, China: a cross-sectional study. BMC Public Health. (2025) 25:2024. doi: 10.1186/s12889-025-22622-6, PMID: 40450225 PMC12125889

[29] Adewuya AO Oladipo O Ajomale T Adewumi T Momodu O Olibamoyo O. Epidemiology of depression in primary care: Findings from the Mental Health in Primary Care (MeHPriC) project, Lagos, Nigeria. Int J Psychiatry Med. (2022) 57:6−20. doi: 10.1177/0091217421996089, PMID: 33573444

[30] Denche-Zamorano Á Ajenjo-Gomez D Pereira-Payo D Galán-Arroyo C Vega-Muñoz A Contreras-Barraza N. Physical activity frequency and depression in the spanish population. Int J Environ Res Public Health. (2022) 19:14704. doi: 10.3390/ijerph192214704, PMID: 36429424 PMC9691247

[31] Noble RE. Depression in women. Metabolism. (2005) 54:49−52. doi: 10.1016/j.metabol.2005.01.014, PMID: 15877314

[32] Nillni YI Rasmusson AM Paul EL Pineles SL. The impact of the menstrual cycle and underlying hormones in anxiety and PTSD: what do we know and where do we go from here? Curr Psychiatry Rep. (2021) 23:8. doi: 10.1007/s11920-020-01221-9, PMID: 33404887 PMC8819663

[33] Alblooshi S Taylor M Gill N. Does menopause elevate the risk for developing depression and anxiety? Results from a systematic review. Australas Psychiatry. (2023) 31:165−73. doi: 10.1177/10398562231165439, PMID: 36961547 PMC10088347

[34] Fava GA Sonino N Morphy MA. Major depression associated with endocrine disease. Psychiatr Dev. (1987) 5:321−48. 3328199

[35] Civieri G Abohashem S Grewal SS Aldosoky W Qamar I Hanlon E. Anxiety and depression associated with increased cardiovascular disease risk through accelerated development of risk factors. JACC Adv. (2024) 3:101208. doi: 10.1016/j.jacadv.2024.101208, PMID: 39238850 PMC11375258

[36] Masson E. EM-consulte. In: La prévalence et les facteurs associés à la dépression et à l’anxiété chez les patients ayant eu un infarctus du myocarde, Tunis, Tunisie. Available online at: https://www.em-consulte.com/article/1075153/la-prevalence-et-les-facteurs-associes-a-la-depres.

[37] Thomas SA Chapa DW Friedmann E Durden C Ross A Lee MCY. Depression in patients with heart failure: prevalence, pathophysiological mechanisms, and treatment. Crit Care Nurse. (2008) 28:40−55. doi: 10.4037/ccn2008.28.2.40, PMID: 18378727

[38] Glassman AH. Depression and cardiovascular comorbidity. Dialogues Clin Neurosci. (2007) 9:9−17. doi: 10.31887/DCNS.2007.9.1/ahglassman, PMID: 17506222 PMC3181839

[39] Anyfanti P Gavriilaki E Pyrpasopoulou A Triantafyllou G Triantafyllou A Chatzimichailidou S. Depression, anxiety, and quality of life in a large cohort of patients with rheumatic diseases: common, yet undertreated. Clin Rheumatol. (2016) 35:733−9. doi: 10.1007/s10067-014-2677-0, PMID: 24859781

[40] Gunn JM Ayton DR Densley K Pallant JF Chondros P Herrman HE. The association between chronic illness, multimorbidity and depressive symptoms in an Australian primary care cohort. Soc Psychiatry Psychiatr Epidemiol. (2012) 47:175−84. doi: 10.1007/s00127-010-0330-z, PMID: 21184214

[41] Stein MB Cox BJ Afifi TO Belik SL Sareen J. Does co-morbid depressive illness magnify the impact of chronic physical illness? A population-based perspective. Psychol Med. (2006) 36:587−96. doi: 10.1017/S0033291706007239, PMID: 16608557

[42] Read JR Sharpe L Modini M Dear BF. Multimorbidity and depression: A systematic review and meta-analysis. J Affect Disord. (2017) 221:36−46. doi: 10.1016/j.jad.2017.06.009, PMID: 28628766

[43] Camara A Sobngwi E Moussa Baldé N Baldé S Oumar Barry T Malal Bah M. P95 - Anxiété et dépression : fréquences et facteurs associés chez 435 diabétiques suivis en Guinée. Diabetes Metab. (2011) 37:A57−8. doi: 10.1016/S1262-3636(11)70721-2

[44] Sareen J Jacobi F Cox BJ Belik SL Clara I Stein MB. Disability and poor quality of life associated with comorbid anxiety disorders and physical conditions. Arch Intern Med. (2006) 166:2109−16. doi: 10.1001/archinte.166.19.2109, PMID: 17060541

[45] Torta RGV Ieraci V. Depressive disorders and pain: A joint model of diagnosis and treatment. J Pain Relief. (2013) 4:1−14. doi: 10.4172/2167-0846.1000S2-003

[46] de Lima CdeA de Oliveira RC de Oliveira SAG da Silva MAS Lima AdeA Andrade MS. Quality of life, anxiety and depression in patients with chronic obstructive pulmonary disease. Rev Bras Enferm. (2020) 73 Suppl 1:e20190423. doi: 10.1590/0034-7167-2019-0423, PMID: 32667477

[47] Chaudri NA. Adherence to long-term therapies evidence for action. Ann Saudi Med. (2004) 24:221−2. doi: 10.5144/0256-4947.2004.221

[48] Fredericksen RJ Gibbons L Brown S Edwards TC Yang FM Fitzsimmons E. Medication understanding among patients living with multiple chronic conditions: Implications for patient-reported measures of adherence. Res Soc Adm Pharm RSAP. (2018) 14:540−4. doi: 10.1016/j.sapharm.2017.06.009, PMID: 28651924 PMC5738290

[49] DiMatteo MR Lepper HS Croghan TW. Depression is a risk factor for noncompliance with medical treatment: meta-analysis of the effects of anxiety and depression on patient adherence. Arch Intern Med. (2000) 160:2101−7. doi: 10.1001/archinte.160.14.2101, PMID: 10904452

[50] Jiakponna EC Agbomola JO Ipede O Karakitie LO Ogunsina AJ Adebayo KT. Psychosocial factors in chronic disease management: Implications for health psychology. Int J Sci Res Arch. (2024) 12:117−28. doi: 10.30574/ijsra.2024.12.2.1219

[51] Hovinga CA Asato MR Manjunath R Wheless JW Phelps SJ Sheth RD. Association of non-adherence to antiepileptic drugs and seizures, quality of life, and productivity: survey of patients with epilepsy and physicians. Epilepsy Behav EB. (2008) 13:316−22. doi: 10.1016/j.yebeh.2008.03.009, PMID: 18472303

[52] Religioni U Barrios-Rodríguez R Requena P Borowska M Ostrowski J. Enhancing therapy adherence: impact on clinical outcomes, healthcare costs, and patient quality of life. Med (Mex). (2025) 61:153. doi: 10.3390/medicina61010153, PMID: 39859135 PMC11766829

